# In Vitro Reversible and Time-Dependent CYP450 Inhibition Profiles of Medicinal Herbal Plant Extracts *Newbouldia laevis* and *Cassia abbreviata*: Implications for Herb-Drug Interactions

**DOI:** 10.3390/molecules21070891

**Published:** 2016-07-07

**Authors:** Nicholas Ekow Thomford, Kevin Dzobo, Denis Chopera, Ambroise Wonkam, Alfred Maroyi, Dee Blackhurst, Collet Dandara

**Affiliations:** 1 Division of Human Genetics, Department of Pathology & Institute for Infectious Disease and Molecular Medicine, Faculty of Health Sciences, University of Cape Town, Anzio Road, Observatory 7925, Cape Town, South Africa; thmnic023@myuct.ac.za (N.E.T.); ambroise.wonkam@uct.ac.za (A.W.); 2 School of Medical Sciences, University of Cape Coast, Cape Coast, PMB, Ghana; 3 ICGEB, Cape Town Component, Faculty of Health Sciences, University of Cape Town, Anzio Road, Observatory 7925, Cape Town, South Africa; kd.dzobo@uct.ac.za; 4 Division of Medical Biochemistry, Faculty of Health Sciences, University of Cape Town, Anzio Road, Observatory 7925, Cape Town, South Africa; 5 Division of Medical Virology, University of Cape Town, Anzio Road, Observatory 7925, Cape Town, South Africa; denis.chopera@uct.ac.za; 6 Department of Botany, University of Fort Hare, Private Bag X1314, Alice 5700, South Africa; amaroyi@ufh.ac.za; 7 Division of Chemical Pathology, University of Cape Town, Anzio Road, Observatory 7925, Cape Town, South Africa; dee.blackhurst@uct.ac.za

**Keywords:** time-dependent inhibition (TDI), CYP450, IC_50_, *Newbouldia laevis*, *Cassia abbreviata*, rCYP

## Abstract

This study evaluated the effects of *Newbouldia laevis* and *Cassia abbreviata* extracts on CYP450 enzyme activity. Recombinant CYP450 enzyme and fluorogenic substrates were used for evaluating inhibition, allowing the assessment of herb–drug interactions (HDI). Phytochemical fingerprinting was performed using UPLC-MS. The herbal extracts were risk ranked for HDI based on the IC_50_ values determined for each CYP enzyme. *Newbouldia laevis* inhibited CYP1A2, CYP2C9, and CYP2C19 enzyme activities with K_i_ of 2.84 µg/mL, 1.55 µg/mL, and 1.23 µg/mL, respectively. *N*. *laevis* exhibited a TDI (4.17) effect on CYP1A2 but not CYP2C9 and CYP2C19 enzyme activities. *Cassia abbreviata* inhibited CYP1A2, CYP2C9, and CYP2C19 enzyme activities showing a K_i_ of 4.86 µg/mL, 5.98 µg/mL, and 1.58 µg/mL, respectively. TDI potency assessment for *Cassia abbreviata* showed it as a potential TDI candidate (1.64) for CYP1A2 and CYP2C19 (1.72). UPLC-MS analysis showed that *Newbouldia laevis* and *Cassia abbreviata* possess polyphenols that likely give them their therapeutic properties; some of them are likely to be responsible for the observed inhibition. The observations made in this study suggest the potential for these herbal compounds to interact, especially when co-administered with other medications metabolized by these CYP450 enzymes.

## 1. Introduction

African populations have used herbal medicines for many centuries. Knowledge regarding specific plants and parts of these plants and their function in disease treatment is traditionally passed on from one generation to the next. With the boom in conventional therapeutic drugs, the utilization of herbal remedies sometimes in combination with other medications has increased tremendously over the last decade [1,2]. It has been demonstrated over the years that herbal remedies possess great therapeutic potential in managing and treating a number of diseases including malaria, hypertension, HIV/AIDS, and cancer. The use of herbal remedies is especially relevant in developing countries [3,4] due to their affordability and accessibility compared to orthodox health interventions. Evidence available shows that there are potential interactions between drugs and herbs, some with fatal clinical outcomes [5,6,7]. Despite these fatal herb-drug clinical outcomes, there is still limited information on monitoring herb–drug interactions [8].

*Newbouldia laevis* (family Bignoniaceae) and *Cassia abbreviata* (family Fabaceae) are medicinal herbal plants that are used in Western and Southern African countries for the treatment and management of many conditions. *Newbouldia laevis*, also known as the tree of life or boundary tree, is a shrub or small tree that grows in West African countries such as Ghana, Nigeria, and Benin. Local names for this shrub include Faangum in Cameroon, Sasanemasa/Sesemasa or Esisimansa in Ghana, Lifui in Togo, and Akoko in Nigeria. The leaves, stems, and roots are used to treat conditions such as malaria, convulsions, cancer, dyslipidemia, diabetes, hemorrhoids, constipation, peptic ulcer, and cough [9,10]. *Cassia abbreviata* is a medium-sized tree that grows in most parts of Africa, especially utilized in Southern African countries such as Botswana, South Africa, Uganda, Zambia, and Zimbabwe. Local names for this shrub include Muremberembe in Zimbabwe, Mahemba in Tanzania, and Monepenepe in Botswana and South Africa. The leaves, stems, and roots are used to treat and/or manage a number of diseases such as malaria, diabetes, ulcers, headaches, diarrhoea, constipation, and skin diseases [11,12], and are believed to possess anti-HIV activity [13].

Despite the advances made in Western medicine, there are many diseases with no cure and so herbal medicines become the default treatment option. However, in many cases, herbal medicines are used concurrently with conventional drugs, raising the risk for adverse drug reactions (ADRs) through the alteration of the pharmacokinetic and pharmacodynamics profiles through herb–drug interactions (HDI). Drug-metabolizing enzymes (DMEs) play a very important role in the metabolism of xenobiotics including therapeutic drugs. The cytochrome P450 (CYP450) family of enzymes especially plays an important role in the biotransformation of about 70% of xenobiotics and other endogenous substances [14]. The composition of herbal medicines is complicated as they contain a number of different phytochemicals and other substances that give them their therapeutic potential. Notwithstanding the complicated composition of herbal medicines, most of their metabolism is also dependent on DMEs, including the CYP450 enzymes, which might affect the metabolism of other administered drugs, leading to potentially increased risk of HDI [15,16]. There are more than 57 cytochrome P-450 genes identified in humans, with the CYP1, CYP2, and CYP3 families contributing to most of the biotransformation of xenobiotics (including therapeutic drugs) [17,18]. This study concentrated on investigating the effects of potential interaction between extracts from *Newbouldia laevis* and *Cassia abbreviata* on CYP1A2, CYP2C9, and CYP2C19 activities.

CYP1A2 is responsible for the metabolism of drugs including theophylline, caffeine, imipramine, paracetamol, and phenacetin [19]. Among the substrates of CYP2C9 is the anticoagulant warfarin, non-steroidal anti-inflammatory drugs (NSAIDs) such as ibuprofen, diclofenac, and naproxen, the hypoglycemic agent tolbutamide, phenytoin, and the angiotensin-II receptor antagonist losartan. CYP2C19 metabolizes a number of commonly used drugs including benzodiazepine diazepam, the proton pump inhibitor omeprazole, propranolol, and the antidepressant amitriptyline [19].

Despite the long-term use of *Newbouldia laevis* and *Cassia abbreviata* by several African populations for various ailments, their effects on cytochrome P450 and information on their potential to cause herb-drug interaction (HDI) by inhibition of cytochrome P450 has not been reported. This study therefore investigated the inhibition profiles of extracts from two medicinal plants, *Newbouldia laevis* and *Cassia abbreviata*, on three major CYP450 enzymes, CYP1A2, CYP2C9, and CYP2C19, using recombinant human CYP450 enzymes. Enzyme kinetic studies were carried out to determine the inhibitory mechanisms of *Newbouldia laevis* and *Cassia abbreviata*. The potential of these two herbal plants to cause HDI was also evaluated. Phytofingerprinting was performed in order to evaluate the profile of possible phytocompounds in both *Newbouldia laevis* and *Cassia abbreviata*.

## 2. Results

### 2.1. Inhibition and TDI Potency

**Figure 1:**
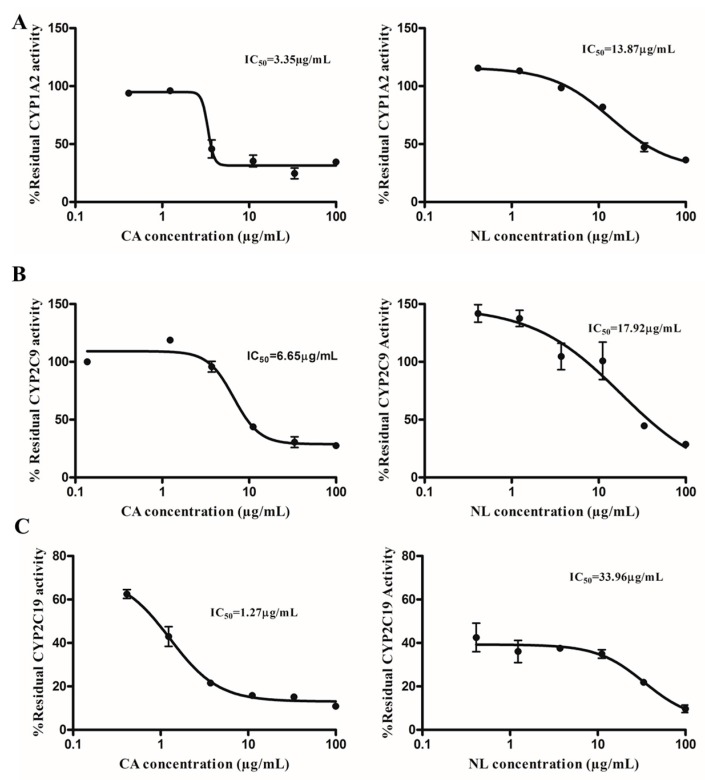
Inhibition profiles (IC_50_ plots) of extracts from *Newbouldia laevis* (NL) and *Cassia abbreviata* (CA) on CYP enzymes. (**A**): CYP1A2; (**B**): CYP2C9 and (**C**): CYP2C19.

The potency of extracts from *Newbouldia laevis* (NL) and *Cassia abbreviata* (CA) to cause inhibition of CYP1A2, CYP2C9, and CYP2C19 activity using the rCYP enzymes was investigated. Figure 1 shows the inhibition profiles of NL and CA on CYP1A2, CYP2C9, and CYP2C19. IC_50_ values are calculated as explained in Section 4. It was observed that the CA extracts caused strong inhibition of CYP1A2 (3.35 ± 0.14 µg/mL), CYP2C9 (6.65 ± 0.34 µg/mL), and CYP2C19 (1.27 ± 0.14 µg/mL), depicting its potency as an inhibitor of the CYP enzymes. NL extracts also caused strong inhibition of CYP1A2 (13.87 ± 0.47 µg/mL) and CYP2C9 (17.92 ± 1.58 µg/mL) and moderate inhibition of CYP2C19 (33.96 ± 1.96 µg/mL). Positive inhibitors of CYP1A2, α-naphthoflavone (0.09 ± 0.01 µg/mL), CYP2C9, sulphaphenazole (0.98 ± 0.12 µg/mL), and CYP2C19, miconazole (0.81 ± 0.02 µg/mL) were used for validation.

**Figure 2:**
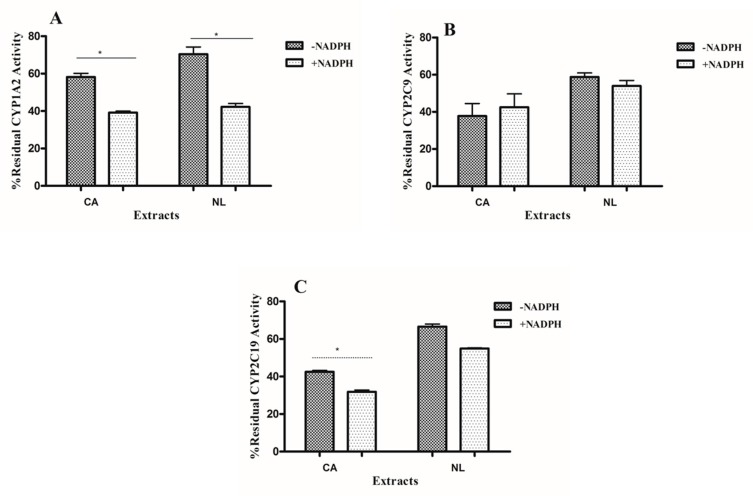
Inhibitory effects of 50 µg/mL concentration of *Newbouldia laevis* (NL) and *Cassia abbreviata* (CA) after pre-incubation with NADPH. (**A**): CYP1A1; (**B**): CYP2C9 and (**C**): CYP2C19, * Significant *p* value.

Single point concentrations of the plant extracts at 50 µg/mL were pre-incubated with or without NADPH and the percentage residual activity compared (Figure 2). Medicinal plant extracts that were pre-incubated with NADPH showed a significant reduction (*p* < 0.05) in percent activity of CYP1A2, depicting its TDI potency without the possibility of enzyme recovery; this was not observed for CYP2C9 and CYP2C19 enzyme activities.

**Figure 3:**
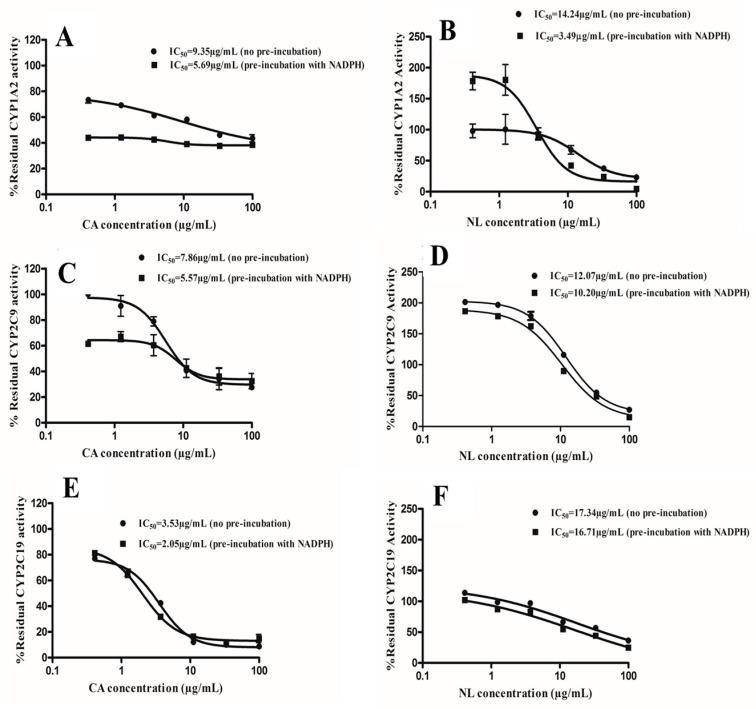
IC_50_ curve shift for time-dependent inhibition (TDI) determination. *Newbouldia laevis* (NL) and *Cassia abbreviata* (CA) at various concentrations were incubated with or without NADPH for 30 min. Percentage residual activity for no pre-incubation (closed circles) and 30 min pre-incubation (closed squares) is shown. (**A**,**B**): CYP1A2; (**C**,**D**): CYP2C9 and (**E**,**F**): CYP2C19.

**Figure 4:**
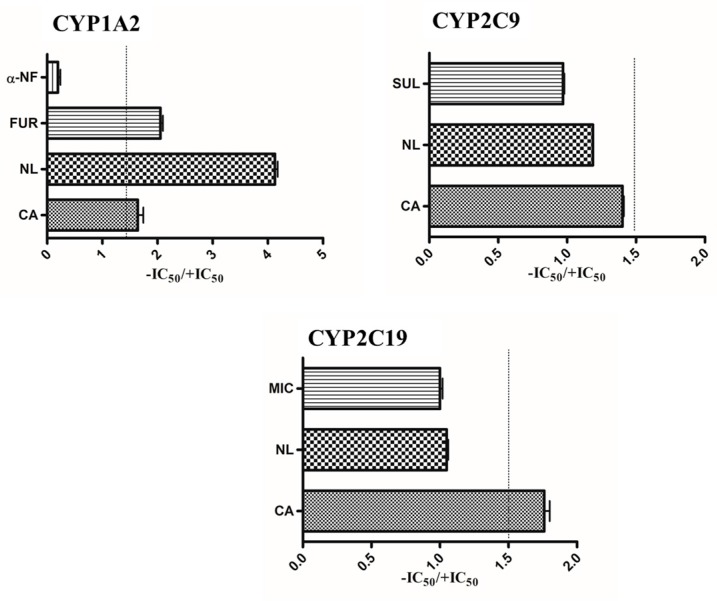
Time-dependent inhibition (TDI) classification of medicinal plant extracts on CYP1A2, CYP2C9, and CYP219 activity based on the IC_50_ curve shift. Extracts with a ratio of ≥1.5 were classified as potential TDI: α-NF—Alpha naphtoflavone; FUR—Furafylline; SUL—Sulphaphenazole; MIC—Miconazole.

The IC_50_ curve shift approach used to assess the TDI potency of the extracts was adopted according to Berry and Zhao [20]. The IC_50_ curve shift method employs a fold shift decrease in IC_50_ values as a result of pre-incubating with or without NADPH. The TDI curves of the extracts of NL and CA on CYP1A2, CYP2C9, and CYP2C19 are shown in Figure 3. A fold change, which leads to a decrease in IC_50_, is used to categorize a plant extract as a potential TDI. Plant extracts that give a ratio (IC_50_ from pre-incubation without NADPH/IC_50_ from pre-incubation with NADPH) of ≥1.5 were classified as a positive TDI, as shown in Figure 4.

Extracts of NL and CA showed a TDI potency (dashed line) with an IC_50_ ratio of greater than 1.5 on CYP1A2 (Figure 4). The assay was validated with furafylline (a known CYP1A2 TDI compound) and α-naphthoflavone (no known TDI effect on CYP1A2). NL and CA extracts did not show any TDI effects on CYP2C9. NL did not show any TDI effects on CYP2C9; however, CA exhibited a TDI effect on CYP2C19, as shown in Figure 4.

### 2.2. Kinetics of Inactivation

**Figure 5:**
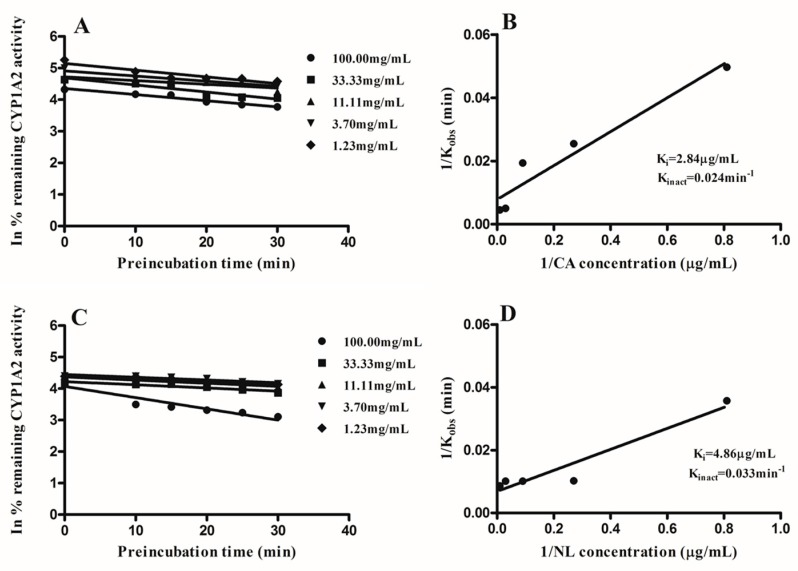
Double reciprocal plots of K_obs_ and inhibitor concentrations were plotted to determine the inactivation parameters K_i_ and K_inact_ on CYP1A2 activity by *Newbouldia laevis* (NL) and *Cassia abbreviata* (CA). (**A**,**B**) represent K_obs_ and double reciprocal curve plots CA, respectively; (**C**,**D**) represent K_obs_ double reciprocal curve plot NL, respectively.

**Figure 6:**
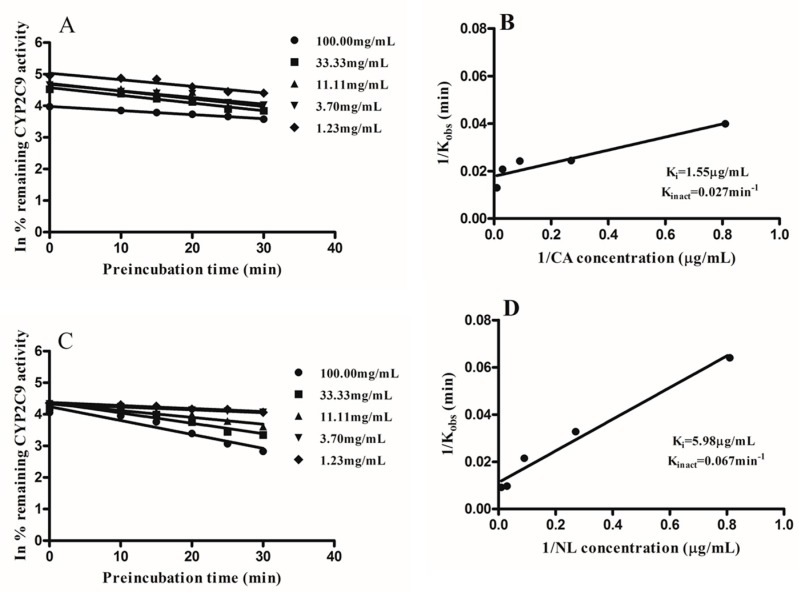
Double reciprocal plots of K_obs_ and inhibitor concentrations were plotted to determine the inactivation parameters K_i_ and K_inact_ on CYP2C9 activity by *Newbouldia laevis* (NL) and *Cassia abbreviata* (CA). (**A**,**B**) represent K_obs_ and double reciprocal curve plots CA, respectively; (**C**,**D**) represent K_obs_ double reciprocal curve plot NL, respectively.

**Figure 7:**
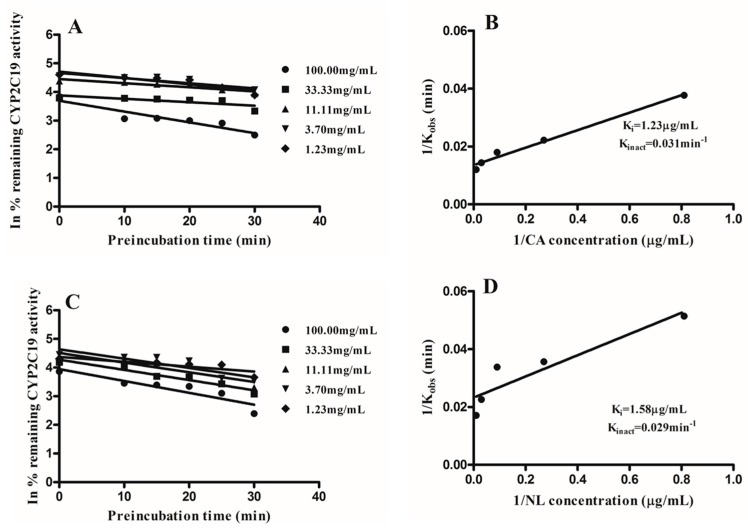
Double reciprocal plots of K_obs_ and inhibitor concentrations were plotted to determine the inactivation parameters K_i_ and K_inact_ on CYP2C19 activity by *Newbouldia laevis* (NL) and *Cassia abbreviata* (CA). (**A**,**B**) represent K_obs_ and double reciprocal curve plots CA, respectively; (**C**,**D**) represent K_obs_ double reciprocal curve plot NL, respectively.

CYP1A2, CYP2C9, and CYP2C19 activity were inactivated in a reversible and time-dependent manner by extracts of NL and CA. Linear regression analysis of the time course was used to determine the K_obs_ values at various concentrations as seen in Figure 5, Figure 6 and Figure 7. A double reciprocal plot of K_obs_ values and inactivator concentrations was used to determine the inactivation constants K_i_ and K_inact_. The K_i_ and K_inact_ values for NL and CA for the various CYPs under consideration are shown in Figure 5, Figure 6 and Figure 7.

### 2.3. Phytofingerprinting by UPLC-MS

**Figure 8:**
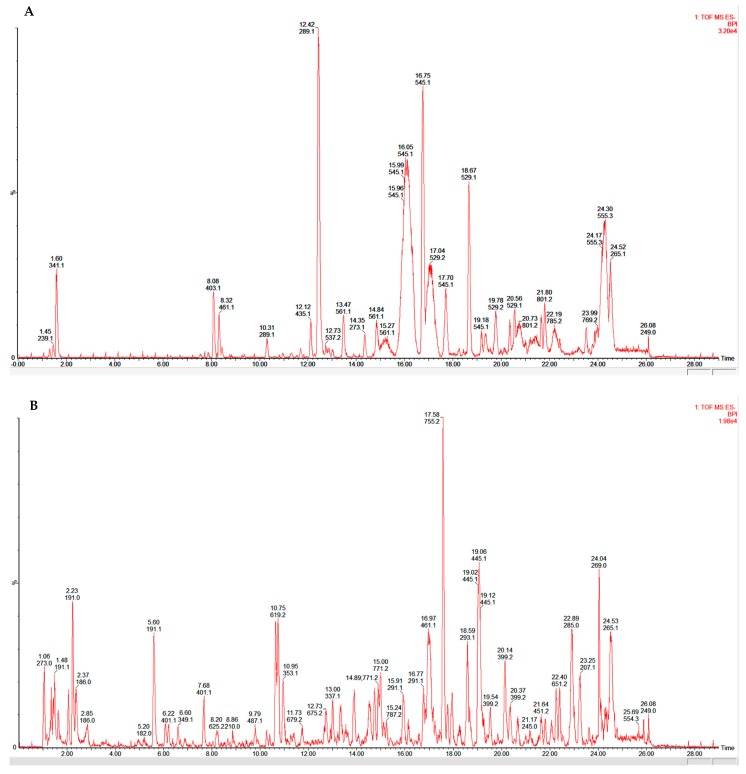
Profiles of crude extracts of *Cassia abbreviata* (CA) and *Newbouldia laevis* (NL) after UPLC-MS analysis. (**A**,**B**) represent chromatograms for CA and NL respectively.

**Figure 9:**
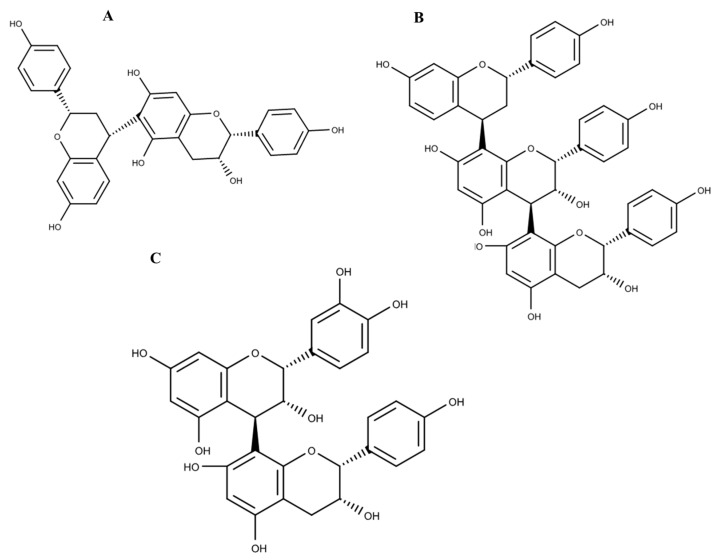
Structures of some of the proposed compounds identified from crude herbal extract of *Cassia abbreviata:* (**A**) Cassiaflavan-(4α→6) epiafzelechin; (**B**) Cassiaflavan-(4β→8) epiafzelechin; (**C**) Epicatechin-(4β→8) epiafzelechin.

**Figure 10:**
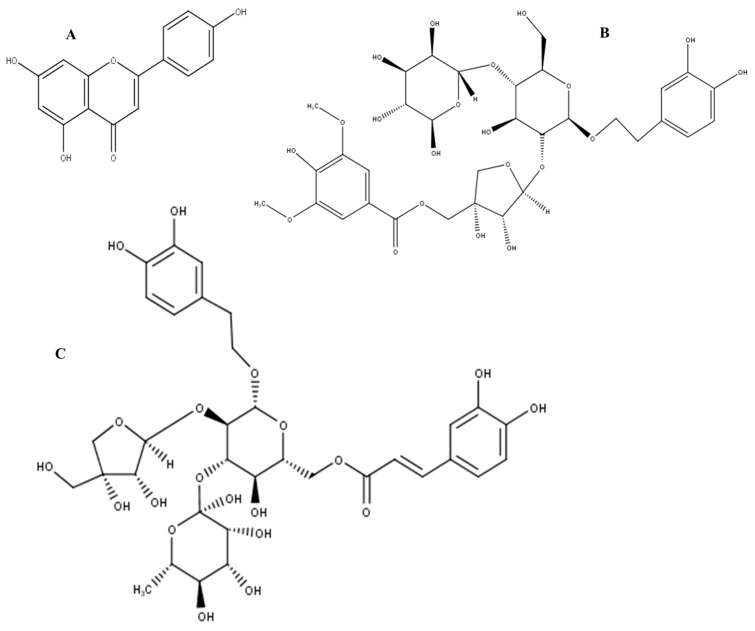
Structures of some of the proposed compounds identified from crude herbal extracts of *Newbouldia laevis*: (**A**) Apigenin; (**B**) Newbouldiside A; (**C**) Luteoside B.

A non-targeted approach was used in order to provide information about all possible metabolites present in the analysed samples. The gradient parameters were adjusted by systematically changing the percentage of solvents to be able to determine as many compounds as possible. The chromatograms obtained (Figure 8) showed the retention times of the various compounds that were found in the extracts. Using fragmentation data, and available databases, possible compounds that might be found in the extracts were proposed for CA (Table 1) and NL (Table 2). Selected structures of these polyphenols are shown in Figure 9 and Figure 10 for *Cassia abbreviata* and *Newbouldia laevis* respectively.

### 2.4. Prediction of in Vivo Herb–Drug Interaction for IC_50_

It is becoming a common occurrence for in vitro data from HDI studies to be used to predict in vivo HDI risk [21,22,23]. Therefore, in this study, the putative GIT concentration that serves as the inhibitor concentration was calculated as shown in Table 3. Since the intestinal absorption and plasma concentration of each test compound are not known and with the knowledge that herbal extracts have different bioavailability [24], the bioavailable concentration was estimated using the % yield (weight extracted powdered material/weight of original starting material) to give the soluble extract available in the GIT tract.

With this knowledge, differential bioavailable concentration was calculated as seen in Table 3. The IC_50_ values were then compared to the calculated estimated bioavailable concentration as reported previously [23] and predictions made (Table 4). It was observed that all the IC_50_ values obtained were three times lower than the estimated bioavailable concentration and therefore adequate amounts that may enter the hepatic portal vein can interact with CYP1A2, CYP2C9, and CYP2C19 enzymes.

## 3. Discussion

The therapeutic efficacy of medicinal herbal plants is well acknowledged in both developed and developing countries. Medicinal plants from different countries mostly lack valid documentation on their safety, efficacy, manufacturing standards, and quality control. Given the varied amounts of compounds found in medicinal herbal plants, standardization parameters and development of marker profiles of commonly used medicinal plants are very important for maintaining the quality of herbal remedies and also provide knowledge on the optimal concentrations of the various bio-constituents [25,26]. Despite being natural, medicinal plant extracts are not free from risks and interactions with other drugs and herbs, which may lead to significant public health consequences including the possibility of being fatal [5,6]. Traditionally, whole plants or mixtures of medicinal plants are used rather than isolated compounds from these plants. Evidence indicates that crude plant extracts often have greater in vitro and/or in vivo therapeutic activity than isolated constituents at an equivalent dose. The aim of this study therefore was to evaluate the CYP450 inhibition activities of *Newbouldia laevis* and *Cassia abbreviata* extracts commonly used in West and Southern Africa for the treatment and/or management of common communicable and non-communicable diseases. It was observed that extracts from these two plants had differential inhibitory effects on CYP1A2, 2C9, and 2C19. This could be used for initial assessment of the potential risk of HDI, which would bring awareness and educate the public and/or patients on the dangers of co-use of herbal medicinal remedies and conventional medications.

Drug interactions can result in clinical fatalities; thus, in the drug discovery process, guidelines and opinions have been published by the FDA, EMA, and pharmaceutical industries [27,28] for the conducting of drug–drug interaction (DDI) studies to help target drugs that are likely to cause interactions at the early stages so that such entities can be either discontinued or modified before further processing. The potential interactions of herbal remedies with prescribed or over-the-counter drugs has been a major safety concern for clinicians and public health practitioners, as such interactions are difficult to predict and also, generally, there is a lack of available information on the composition of herbs and their pharmacological actions [29]. With the tremendous surge in the acceptance of and public interest in herbal medicinal remedies, researchers are adopting the guidelines and opinions proposed for DDI studies to study HDI. This is helpful with labelling purposes for commercially available herbal products and also to caution patients from combining herbal remedies with known conventional medications [30]. There is limited information currently available regarding the potential of *Newbouldia laevis* and *Cassia abbreviata* to interact with clinically prescribed drugs. This is a safety issue as these herbal plant remedies are commonly used by certain populations. This study therefore looked at the drug interaction potential of *N. laevis* and *C. abbreviata* extracts on three major cytochrome P450 enzymes, CYP1A2, CYP2C9, and CYP2C19. Typically, new chemical entities (NCE) are assessed for their potential to cause CYP-mediated drug interaction based on a single concentration screening and later a detailed classification involving IC_50_, TDI, and inactivation constants like K_i_ and K_inact_.

Crude *N. laevis* and *C. abbreviata* extracts were used in this study to mimic the way patients take these herbal remedies and also the mode of extraction simulated to show the indigenous mode of extraction. *Newbouldia laevis* extract showed a strong reversible inhibitory effect on CYP1A2 and CYP2C9 enzyme activities whilst exhibiting a moderate reversible inhibition on CYP2C19. *Cassia abbreviata* extract showed a strong reversible inhibitory effect on CYP1A2, CYP2C9, and CYP2C19 enzyme activities. The implication is that there is a possibility of enzyme recovery should the herbal extracts inactivate these CYPs.

Time-dependent inhibition of CYP450 enzymes that is caused by NCEs is of great concern because such compounds can cause clinically relevant DDI or HDI [31]. The TDI of a CYP enzyme refers to the change in inhibition potency of a NCE as a result of a dosing period that leads to formation of inhibitory metabolites and mechanism-based inhibition [32]. The use of the IC_50_-shift approach as an initial step for TDI assessment has been recommended by the Pharmaceutical Research and Manufacturers Association (PhRMA) [27,28,29,30,31,32], where HLMs or recombinant CYPs are used to evaluate the increase in potency of a NCE to cause CYP-inhibition when the chemical entity is pre-incubated with NADPH [20,33]. This approach was used to assess the TDI potency of *Newbouldia laevis* and *Cassia abbreviata* extracts. Though the IC_50_-shift approach is recommended, there is a challenge with the interpretation of the criterion used to define a chemical as a potential TDI. PhRMA recommends the criterion of 1.2- to 3-fold decrease [32] for classification of potential TDI candidates, whilst Berry and Zhao [20] have adopted a criterion of ≥1.5 as being significant for classification for TDI candidates. This study therefore adopted the Berry and Zhao approach of setting the TDI criterion at ≥1.5. As seen in Figure 4, *Newbouldia laevis* and *Cassia abbreviata* showed TDI potencies of 4.08 and 1.64, respectively, on CYP1A2. TDI potency of 0.15 and 2.01 was observed for α-naphthoflavone and furafylline known non-TDI and TDI compounds respectively. *Newbouldia laevis* and *Cassia abbreviata* extracts did not exhibit any TDI potency on CYP2C9 enzyme with values of 1.18 and 1.41, respectively, implying they were not potential CYP2C9 TDI candidates. Although *Newbouldia laevis* extract exhibited a non-TDI effect (1.03) on the CYP2C19 enzyme, *Cassia abbreviata* extract exhibited a TDI effect on the CYP2C19 enzyme with a ratio of 1.72. Sulphaphenazole and miconazole, which are non-TDI compounds of CYP2C9 and CYP2C19, were used to validate the assay, respectively.

The differential reversible and irreversible inhibition of CYP1A2, CYP2C9, and CYP2C19 enzymes by *Newbouldia laevis* and *Cassia abbreviata* extracts implies that patients who are on prescribed drugs that have narrow therapeutic ranges (for example, theophylline, caffeine, imipramine, paracetamol, phenacetin-CYP1A2, warfarin, non-steroidal anti-inflammatory drugs (NSAIDs), tolbutamide, phenytoin, losartan-CYP2C9, diazepam, omeprazole, propranolol, amitriptyline-CYP2C19, or over-the-counter drugs that are metabolized by similar CYP450 enzymes) stand a risk of having HDI, which can lead to clinical consequences. The TDI effects of these herbals is felt when patients chronically use them because sufficient intermediary metabolites may be generated and this can be deleterious to consumers concomitantly taking conventional medications, as stated earlier.

Single point screening for the effects of pre-incubation with NADPH was performed on CYP1A2, CYP2C9, and CYP2C19. Data showed significant effects of pre-incubation of *Newbouldia laevis* extracts on CYP1A2 enzyme activity but not CYP2C9 and CYP2C19 enzyme activities. *Cassia abbreviata* extracts, however, exhibited significant effects on CYP1A2 and CYP2C19 enzyme activities after pre-incubation with NADPH but not CYP2C9 enzyme activity. These results combined with the TDI potencies imply that these herbs’ inhibitory effects may either be increased or reduced after pre-incubation. The weakening inhibition potency could be due to recovery of enzyme activity since the inhibition potency of the herbs weakens with time regardless of the concentration applied, whilst the increase in inhibitory potency could be due to the interaction of the herb with CYP450 enzymes during pre-incubation.

Following the inactivation of CYP1A2, CYP2C9, and CYP2C19, the inactivation parameters were determined. The kinetic parameters for the inactivation of CYP1A2 by *Newbouldia laevis* and *Cassia abbreviata* extracts were: K_i_; 2.84 µg/mL and 4.86 µg/mL, K_inact_; 0.024 min^−1^ and 0.033 min^−1^, respectively. Inactivation kinetic parameters of CYP2C9 were: K_i_; 1.55 µg/mL and 5.98 µg/mL, K_inact_; 0.027 min^−1^ and 0.067 min^−1^, respectively. Kinetics parameters of inactivation for CYP2C19 were K_i_; 1.23 µg/mL and 1.58 µg/mL, K_inact_; 0.031 min^−1^ and 0.029 min^−1^, respectively. Determination of inactivation parameters gives more information on the type of inactivation, thus allowing patients taking these herbs concomitantly with conventional medication to be aware of the implications.

Herbal medicines consist of multi-phytochemical constituents with different physicochemical properties. The risk of HDI of orally administered herbal remedies depends on the bioavailable fraction of the phyto-constituents [34], which may or may not permeate through membrane barriers. Due to the multi-phyto nature of herbals, their bio-availabilities are variable depending on the constituents [24]. Prediction of in vivo HDI using in vitro results [21,23,35,36] gives preliminary information that can caution patients to be wary of combination therapies of herbals with conventional medications and also for pharmaceutical companies and researchers to get baseline information guiding further investigation. For herbal extracts that are already in use, HDI studies allow assessment of risk, helping with the design of in vivo HDI studies and revision of product labels to highlight the risk of co-use of these herbs with conventional medication, as in the case of Saint John′s Wort and the protease inhibitor indinavir [37]. This study assessed the risk of HDI on CYP1A2, CYP2C9, and CY2C19 activities and predicted these herbals can cause HDIs in vivo to a significant extent, with the assumption that at least one phyto-constituent is absorbed to cause HDI.

Profiling of herbal extracts for the phyto-constituents is a critical step in elucidating the possible mechanisms for HDI potential. Based on the UPLC-MS data obtained, the crude extracts of *Newbouldia laevis* and *Cassia abbreviata* are composed of several different phenolic compounds. The UPLC-MS profiles indicated the presence of at least 10 polyphenolic candidates including catechins and potent antioxidant flavonoids, which likely give these herbs their therapeutic effects. Epidemiological studies of polyphenols suggest that consumption of polyphenol-rich herbs and beverages may contribute to the prevention of diseases including cancers, cardiovascular diseases, osteoporosis, neurodegenerative diseases, and diabetes [38,39]. However, some of these polyphenols are known to inhibit CYP450 [40,41,42,43]. It is therefore possible that the interactions observed in this study may be related to some of the constituents that are in these herbs.

## 4. Materials and Methods

### 4.1. Chemicals and Reagents

Inhibition and kinetic reactions were carried out in Black costar 96-well plates bought from Thermo Fischer Scientific (Pittsburgh, PA, USA). α-naphthoflavone, furafylline, sulphaphenazole, and miconazole were purchased from Sigma-Aldrich (St. Louis, MO, USA). Vivid^®^ Blue screening kits and vivid^®^ substrates, 7-benzyl-oxymethyloxy-3-cyanocoumarin, (BOMCC-cat. no. P2975) and 7-ethoxy-methloxy-3-cyanocoumarin (EOMCC-cat. no. P3024) were obtained from Life Technologies, (Grand Island, NY, USA). CYP2C19 (cat. no. P2864), CYP2C9 (cat. no. P2861), and CYP1A2 (cat. no. P2863) blue screening kit included baculosome (respective isozymes and NADPH-P450 reductase); a regeneration system (glucose-6-phosphate, glucose-6-phosphate dehydrogenase) and NADP^+^ were used for the study. Standards for epicatechin, catechin, chlorogenic acid, caffeic acid, and *p*-coumaric acid for identification and quantification of constituents of herbal extracts were purchased from Chromadex (Wesel, Germany). Ultra-Pure double distilled and deionized water was obtained from Milli-Q plus water purification system (Millipore, Bedford, MA, USA). All reagents were of analytical grade.

### 4.2. Plant Material Extraction

The leaves of *Newbouldia laevis* (UCC/BS/689) and *Cassia abbreviata* (UB/B/151) were obtained with the assistance of traditional herbal practitioners and authenticated by a botanist from the University of Cape Coast herbarium. Voucher specimens were kept at the University of Cape Coast herbarium. The plant material was air-dried at room temperature and ground into a fine powder. Extraction was performed using water to mimic the indigenous mode of extraction. Powdered plant material (10 g) was added to 100 mL of distilled water, heated for one hour at 60 °C, and allowed to extract for 72 h with 24 h decanting of supernatant and refilling with the same volume of distilled water on solid residue. Supernatants were pooled, centrifuged (14,000× *g*, 10 min), and filtered using a filter paper (8 µm, Whatman International LTD, Maidstone, UK). Filtrates were freeze-dried using a Virtis sentry freeze dryer (the Virtis Company, NC, Gardiner, NY, USA) and the dried powder stored in airtight containers at –20°C until subsequent experiments.

### 4.3. CYP450 Inhibition

The determination of reversible inhibition was carried out as reported in a previous study with slight modification [23]. An initial study of two-point screening indicated that 100 µg/mL exhibited the maximum inhibitory effect on CYP1A2, CYP2C9, and CY2C19. Reconstitution of extracts in subsequent experiments were performed with 100 µg/mL concentration as the highest concentration. Briefly, serial dilutions (1:3) of the medicinal plant extracts from a concentration of 100 µg/mL were pre-incubated with a mixture of CYP 450 BACULOSOME^®^ (CYP1A2, CYP2C9, CYP2C19) plus reagent and regeneration system (consisting of glucose-6-phosphate and glucose-6-phosphate dehydrogenase) in Vivid^®^ reaction buffer in black costar 96-well plate for 20 min at 37 °C. The reaction was initiated by the addition of reconstituted substrate as seen in Table 5 and NADP^+^ in Vivid^®^ reaction buffer and incubated for a further 30 min at 37 °C. The reaction was stopped by the addition of ice-cold 20% Tris base/80% acetonitrile (ACN). Enzyme activity was monitored by measuring the formation of fluorescent metabolite at excitation/emission wavelength of 405/460 nm. Fluorescence was measured using a Varian Cary eclipse (SSN instruments, Set Point Technology, South Africa) with Advanced reads software. Conditions for the experiment are shown in Table 5. Suggested standard inhibitors for the CYP enzymes were supplied by the manufacturer.

### 4.4. Determination of Time-Dependent Inhibition (TDI) Potency

The procedure followed was similar to that used for the screening of CYP450 inhibition with a slight modification that included employing the curve shift method by Berry and Zhao [20]. Plant extracts with a starting concentration of 100 µg/mL were serially diluted (1:3) in black costar 96-well plates. The black costar plates were divided into two halves. One half contained a reaction mixture made of BACULOSOME^®^, regeneration system, NADP^+^, and Vivid^®^ reaction buffer, Table 1 and the other half contained BACULOSOME^®^, a regeneration system, and Vivid^®^ reaction buffer (Table 5). The mixture was pre-incubated for 30 min at 37 °C. After the pre-incubation step, reconstituted substrate (Table 5) and NADP^+^ in Vivid^®^ reaction buffer was added to both halves of the plate and incubated for a further 30 min at 37 °C. The reaction was terminated by adding ice-cold 20% Tris base/80% ACN. The activity of the enzymes was monitored by measuring the formation of fluorescent metabolite at the same excitation and emission wavelength as in the CYP450 inhibition determination. Single point NADPH screening with a single concentration of 50 µg/mL was performed as in the TDI determination to assess the significance of the effect of pre-incubating with NADPH at a single concentration.

### 4.5. Estimation of Kinetics of Inactivation

The method used is a slight modification of the CYP450 inhibition and TDI determinations according to the method of Krippendorff et al. [44]. Briefly, serial dilutions of the extracts were added to a mixture of regeneration system, NADP^+^, and Vivid^®^ reaction buffer and pre-warmed. After pre-warming, the reaction was started by adding a mixture of substrate and BACULOSOME^®^ in Vivid^®^ reaction buffer. Fluorescence was measured for each well as indicated previously. However, with this method, instead of using end point measurements as done previously, CYP activity was determined every 5 min up to 30 min after the start of the reaction using an excitation/emission wavelength of 405 nm/460 nm.

### 4.6. UPLS-MS Analysis and Relative Quantification of Phenolic Compounds

Phytofingerprinting was performed on the extracts using a Waters Acquity UPLC system (Waters Corporation, Milford, MA, USA) with an Acquity BEH C18 column (2.1 mm × 100 mm, 1.7 µm particle size) incorporating a binary pump, vacuum degasser, autosampler, column oven, and Micromass Xevo tandem quadrupole mass spectrometric detector (QTOF xevo G2; Waters micromass, Manchester, UK) equipped with ESI probe. Gradient elution was performed at a flow rate of 0.1 mL/min throughout at injection volumes of 10 µL. Gradient parameters were adjusted by systematically changing the percentage organic modifier at initial conditions, and/or the isocratic hold period at initial conditions, and/or gradient steepness. Electrospray mass spectra data were recorded on a negative ionization mode for a mass range *m*/*z* 100 to *m*/*z* 1500 at a collision energy of 50 V. Relative quantification of some constituents of the extracts was performed as stated previously [23]. Standards of epicatechin, catechin, chlorogenic acid, caffeic acid, and *p*-coumaric acid were used to generate calibration curves and quantities derived from the calibration curves. Calibration curve ranges for each reference compound ranged from 0.1 to 100 µg/mL.

### 4.7. Data Analysis

#### 4.7.1. IC_50_ Determination

Data generated were exported into an Excel spreadsheet and the amount of metabolite formed at the various concentrations relative to the control (residual activity) was calculated using the equation below:

(1) $$\%\text{ Residual activity}=\frac{\text{test}-\text{blank}}{\text{control}-\text{blank}}\times 100$$

Percentage residual activity was plotted against the log-transformed concentrations of the extracts and positive inhibitors. Non-linear regression analysis was used for the sigmoid curve fitting and IC_50_ values calculated using GraphPad^®^ Prism version 5.0 (GraphPad^®^ Software Inc., San Diego, CA, USA).

#### 4.7.2. Inactivation Kinetics

The natural logarithm of the percentage remaining activity was plotted against the pre-incubation times at each extract concentration to obtain *K_obs_* (slope) as employed by Awortwe et al. [36]. K_obs_ depicts the rate constant describing the inactivation at each inhibitor (extract) concentration. The K_i_ and K_inact_ values were then determined by a double reciprocal plot of the K_obs_ values and [I] using the equation below:

(2) $$\text{Kobs}=\frac{Kinact\times \left[I\right]}{\text{Ki}+\left[I\right]}$$

where K_inact_ is the maximal rate of inactivation; K_i_ is the inhibitor concentration required for half-maximal inactivation; and [I] is the pre-incubation concentration of inhibitor (extract).

#### 4.7.3. Statistical Analysis

All values are expressed as Mean ± SEM. Comparisons for significance were performed using unpaired *t*-test with *p* < 0.05 considered statistically significant.

## 5. Conclusions

Most patients in developed and developing countries consume herbal medicines for reasons already elucidated in addition to conventional medicines. This study has evaluated the inhibitory potencies of *Newbouldia laevis* and *Cassia abbreviata* on CYP1A2, CYP2C9, and CYP2C19 activities. It is observed that these two herbs caused differential inhibition of the CYPs and are likely to cause HDI. This study gives information on two herbs that have been used for decades and this may be used to caution patients to desist from combining them with other conventional medications.

## Figures and Tables

**Table 1:** Compounds identified in extracts of *Cassia abbreviata* (CA).

Peak	R_t_ (min)	[M − H]^−^	Proposed Formula	Error (ppm)	MS/MS Fragmentation	Proposed Compound
1	1.30	181.0718	C_6_H_14_O_6_	3.3	43, 73, 89, 101, 109	d-mannitol
2	1.60	341.1082	C_12_H_22_O_11_	−0.6	161, 179, 341	Sucrose
3	7.86	431.1177	C_18_H_24_O_12_	−3.0	137, 149, 178	6-β-epiacetylscandoside
4	8.10	403.1237	C_17_H_24_O_11_	−0.7	271	6-β-hydroxygeniposide
5	8.45	417.1392	C_18_H_26_O_11_	−1.2	109, 167, 195	
6	8.32	461.13	C_19_H_25_O_13_	1.5	152, 167, 329	Sibricose A3
7	9.35	491.1425	C_38_H_20_O	−2.2	356, 466, 421, 323, 334, 266, 298, 304	
8	10.29	289.0713	C_15_H_14_O_6_	0.3	179, 188, 205, 245	Catechin
9	12.11	435.1274	C_21_H_24_O_10_	−3.9	289, 313, 342, 311, 393	Epiafzelechin-3-*o*-β-d-glucopyranoside
10	12.73	537.1798	C_22_H_34_O_15_	−3.9		
11	13.48	561.1395	C_30_H_26_O_11_	−0.4	289, 273	Epicatechin-(4β→8) epiafzelechin
12	14.37	273.0765	C_15_H_14_O_5_	0.7	271, 289	(Epi)-Afzelechin
13	16.06	545.1439	C_30_H_26_O_10_	−1.7		Guibourtinidol-(4α→8) epicatechin
14	17.01	529.1502	C_30_H_26_O_9_	0.6		Guibourtinidol-(4α→8) epiafzelechin
15	21.80	801.2195	C_45_H_38_O_14_	1.7		
16	22.19	785.2243	C_45_H_38_O_13_	1.1		Cassiaflavan-(4β→8) epiafzelechin
17	22.41	513.1546	C_30_H_26_O_8_	−0.6		Cassiaflavan-(4α→6) epiafzelechin

**Table 2:** Compounds identified in extract of *Newbouldia laevis* (NL).

Peak	R_t_ (min)	[M − H]^−^	Proposed Formula	Error (ppm)	MS/MS Fragmentation	Proposed Compound
1	2.23	191.0196	C_6_H_8_O_7_	2.1	111, 127, 129	Citric acid
2	5.60	191.0558	C_7_H_12_O_6_	1.0	71, 101, 173	Quinic acid
3	6.60	349.1143	C_14_H_22_O_11_	2.3	440, 518, 591, 600	
4	7.68	401.1087	C_17_H_22_O_11_	0.7	321, 341, 382	10-Dehydrogardenoside
5	7.90	609.2037	C_25_H_38_O_17_	1.0	303, 371, 475, 554, 623	
6	8.20	625.1984	C_25_H_38_O_18_	0.6	359, 499, 515, 593, 661	
7	10.63	619.1869	C_26_H_36_O_17_	–0.8	179, 191, 283, 383	
8	10.95	353.0876	C_16_H_18_O_9_	0.8	179, 191	Chlorogenic acid
9	11.73	679.2097	C_28_H_40_O_19_	1.6	609, 661	4-caffeoylquinic acid
10	12.73	675.1899	C_32_H_36_O_16_	–3.9	173	Elloramycin F
11	13.00	337.0923	C_16_H_18_O_8_	–0.9	119, 163	3-*p*-coumaroylquinic acid
12	13.90	415.1611	C_19_H_28_O_10_	1.7		Aragoside
13	15.00	773.2352	C_34_H_46_O_20_	0.5	135, 147, 161, 179, 417, 452, 591, 619	Newbouldiside A
14	15.91	291.1088	C_12_H_20_O_8_	2.7	105, 135, 147,161	
15	16.97	461.0713	C_21_H_18_O_12_	–1.5	285, 345, 461	Luteolin 7-*O*-glucuronide
16	17.58	755.2393	C_34_H_44_O_19_	–0.8	461, 593	Luteoside B
17	17.94	197.1176	C_11_H_18_O_3_	–1.0	173, 179, 187	
18	19.06	445.0776	C_21_H_18_O_11_	1.1	269, 271	Apigenin 7-*O*-β-glucuronide
19	20.14	399.1649	C_19_H_28_O_9_	–1.5		
20	21.17	245.0456	C_13_H_10_O_5_	2.0	133, 161, 179, 217, 234	Pimpinellin
21	21.80	709.2343	C_33_H_42_O_17_	–0.1	222, 253, 275, 315, 335, 337	
22	22.88	285.0399	C_15_H_10_O_6_	0.0	133, 161, 175, 191, 199, 217	Luteolin
23	23.22	207.0658	C_11_H_12_O_4_	0.5	179	Ferrulic acid methyl ester

**Table 3:** Calculation of herbal medicine concentration in the gut.

Herbal Extracts	% Yield	Recommended Herbal	Putative GIT	Estimated Bioavailable
		Dose (Single; mg)	Concentration (µg/mL)	Concentration (µg/mL)
*Newbouldia laevis*	14.66	200	800	117.28
*Cassia abbreviata*	12.47	200	800	99.76

Note: GIT, Gastrointestinal tract, Putative GIT concentration = dose/ estimated gut fluid volume of 250 mL, estimated bioavailable concentration = (% yield × putative GIT concentration)/100.

**Table 4:** In vivo prediction of HDI from in vitro data for CYP1A2, CYP2C9, and CYP2C19.

Herbal Extracts	Inhibitor	IC_50_ (µg/mL)	Risk of HDI in the Gut *	K_i_	Predicted % Inhibition $\frac{\left[I\right]}{\left[I\right]+Ki}\times 100$
**Concentration (µg/mL)**					
**CYP1A2**					
*Newbouldia laevis*	117.28	13.87	likely	4.86	96.02
*Cassia abbreviata*	99.76	3.35	likely	2.84	97.23
**CYP2C9**					
*Newbouldia laevis*	117.28	17.92	likely	5.98	95.15
*Cassia abbreviata*	99.76	6.22	likely	1.55	98.47
**CYP2C19**					
*Newbouldia laevis*	117.28	33.96	likely	1.58	98.67
*Cassia abbreviata*	99.76	1.27	likely	1.23	98.78

Note: HDI, herb-drug interaction, inhibitor concentration = estimated bioavailable concentration (µg/mL), * the likelihood of a clinically relevant interaction when these herbal extracts are taken is based on the assumption that the % yield serves as the bioavailable fraction, which was used in estimating the bioavailable concentration in the gut and also if there is complete absorption.

**Table 5:** Experimental conditions involved in fluorogenic assay.

Parameter	CYP1A2	CYP2C9	CYP2C19
Substrate (µM)	3 (EOMCC)	10 (BOMCC)	10 (EOMCC)
Enzyme (nM)	5	10	5
Standard inhibitor	α-naphthoflavone/furafylline	Sulphaphenazole	Miconazole
Phosphate buffer	100 mM	100 mM	100 mM
Fluorescence filter	Ex: 405 nm/Em: 460 nm	Ex: 405 nm/Em:460 nm	Ex: 405 nm/Em: 460 nm
Reaction buffer (nM)	200 (Buffer I)	100 (Buffer II)	100 (Buffer II)
Fluorescent product	7-hydroxy-3-cyanocoumarin	7-hydroxycoumarin	7-hydroxy-3-cyanocoumarin
